# Hospital-acquired bloodstream infections in critically ill cirrhotic patients: a post-hoc analysis of the EUROBACT-2 international cohort study

**DOI:** 10.1186/s13613-024-01299-x

**Published:** 2024-05-02

**Authors:** Hannah Wozniak, Alexis Tabah, François Barbier, Stéphane Ruckly, Ambre Loiodice, Murat Akova, Marc Leone, Andrew Conway Morris, Matteo Bassetti, Kostoula Arvaniti, Ricard Ferrer, Liesbet de Bus, Jose Artur Paiva, Hendrik Bracht, Adam Mikstacki, Adel Alsisi, Liana Valeanu, Josef Prazak, Jean-François Timsit, Niccolò Buetti, on behalf of the EUROBACT-2 Study Group, ESICM, ESCMID ESGCIP and the OUTCOMEREA Network

**Affiliations:** 1 Division of Critical Care, Department of Acute Medicine, University Hospital of Geneva, University of Geneva, Geneva, Switzerland; 2 Interdepartmental Division of Critical Care Medicine, University of Toronto, Toronto, Canada; 3 Intensive Care Unit, Redcliffe Hospital, Brisbane, Australia; 4 Queensland Critical Care Research Network (QCCRN), Brisbane, QLD Australia; 5 Queensland University of Technology, Brisbane, QLD Australia; 6 Faculty of Medicine, The University of Queensland, Brisbane, QLD Australia; 7 Service de Médecine Intensive-Réanimation, Centre Hospitalier Régional d’Orléans, Orléans, France; 8 Université de Paris, INSERM, IAME UMR 1137, Paris, 75018 France; 9 ICUREsearch, Biometry, Fontaine, 38600 France; 10 Department of Infectious Diseases, Hacettepe University School of Medicine, Ankara, Turkey; 11 Department of Anesthesiology and Intensive Care Unit, Hospital Nord, Aix Marseille University, Assistance Publique Hôpitaux Universitaires de Marseille, Marseille, France; 12 Division of Anaesthesia, Department of Medicine, University of Cambridge, Addenbrooke’s Hospital, Hills Road, Cambridge, CB2 0QQ UK; 13 Division of Immunology, Department of Pathology, University of Cambridge, Tennis Court Road, Cambridge, Cb2 1QP UK; 14 JVF Intensive Care Unit, Addenbrooke’s Hospital, Cambridge, Hills Road, Cambridge, CB2 0QQ UK; 15 Infectious Diseases Clinic, Department of Health Sciences, University of Genoa and Ospedale Policlinico San Martino, Genoa, Italy; 16 Intensive Care Unit, Papageorgiou University Affiliated Hospital, Thessaloníki, Greece; 17 Intensive Care Department, SODIR-VHIR Research Group, Vall d’Hebron University Hospital, Barcelona, Spain; 18 Department of Critical Care Medicine, Ghent University Hospital, Ghent, Belgium; 19 Department of Internal Medicine and Paediatrics, Faculty of Medicine and Health Sciences, Ghent University, Ghent, Belgium; 20 Intensive Care Medicine Department, Centro Hospitalar Universitário São João (CHUSJ), Porto, Portugal; 21 Department of Medicine, Faculty of Medicine, University of Porto (FMUP), Porto, Portugal; 22 Central Interdisciplinary Emergency Medicine, University Hospital Ulm, Ulm, Germany; 23 Faculty of Health Sciences, Poznan University of Medical Sciences, Poznan, Poland; 24 Department of Anaesthesiology and Intensive Therapy, Regional Hospital in Poznan, Poznan, Poland; 25 ICU Department, Prime Hospital, Dubai, United Arab Emirates; 26 Critical Care Department, Faculty of Medicine, Cairo University, Cairo, Egypt; 27 Cardiac Anesthesiology and Intensive Care Department I, Emergency Institute for Cardiovascular Diseases Prof. Dr. C. C. Iliescu, Bucharest, Romania; 28 Department of Intensive Care Medicine, Bern University Hospital, Inselspital, University of Bern, Bern, Switzerland; 29 Université Paris- Cité, INSERM, IAME UMR 1137, Paris, 75018 France; 30 Medical and Infectious Diseases Intensive Care Unit, AP-HP, Bichat‐Claude Bernard University Hospital, Paris, France; 31 Infection Control Program and World Health Organization Collaborating Centre on Patient Safety, Faculty of Medicine, University Hospitals, University of Geneva, Geneva, Switzerland

## Abstract

**Background:**

Hospital-acquired bloodstream infections are common in the intensive care unit (ICU) and have a high mortality rate. Patients with cirrhosis are especially susceptible to infections, yet there is a knowledge gap in the epidemiological distinctions in hospital-acquired bloodstream infections between cirrhotic and non-cirrhotic patients in the ICU. It has been suggested that cirrhotic patients, present a trend towards more gram-positive infections, and especially enterococcal infections. This study aims to describe epidemiological differences in hospital-acquired bloodstream infections between cirrhotic and non-cirrhotic patients hospitalized in the ICU regarding infection sources, microorganisms and mortality.

**Methods:**

Using prospective Eurobact-2 international cohort study data, we compared hospital-acquired bloodstream infections sources and microorganisms in cirrhotic and non-cirrhotic patients. The association between *Enterococcus faecium* and cirrhosis was studied using a multivariable mixed logistic regression. The association between cirrhosis and mortality was assessed by a multivariable frailty Cox model.

**Results:**

Among the 1059 hospital-acquired bloodstream infections patients included from 101 centers, 160 had cirrhosis. Hospital-acquired bloodstream infection source in cirrhotic patients was primarily abdominal (35.6%), while it was pulmonary (18.9%) for non-cirrhotic (*p* < 0.01). Gram-positive hospital-acquired bloodstream infections accounted for 42.3% in cirrhotic patients compared to 33.2% in non-cirrhotic patients (*p* = 0.02). Hospital-acquired bloodstream infections in cirrhotic patients were most frequently caused by *Klebsiella* spp (16.5%), coagulase-negative Staphylococci (13.7%) and *E. faecium* (11.5%). *E. faecium* bacteremia was more frequent in cirrhotic patients (11.5% *versus* 4.5%, *p* < 0.01). After adjusting for possible confounding factors, cirrhosis was associated with higher *E. faecium* hospital-acquired bloodstream infections risk (Odds ratio 2.5, 95% CI 1.3–4.5, *p* < 0.01). Cirrhotic patients had increased mortality compared to non-cirrhotic patients (Hazard Ratio 1.3, 95% CI 1.01–1.7, *p* = 0.045).

**Conclusions:**

Critically ill cirrhotic patients with hospital-acquired bloodstream infections exhibit distinct epidemiology, with more Gram-positive infections and particularly *Enterococcus faecium*.

**Supplementary Information:**

The online version contains supplementary material available at 10.1186/s13613-024-01299-x.

## Introduction

Hospital-acquired bloodstream infections (HABSIs) are frequent in the intensive care unit (ICU) and are associated with a high mortality [1, 2]. Cirrhotic patients are vulnerable and susceptible to hospital-acquired infections, which can affect as many as 43–59% of patients in the ICU [3–7]. Bloodstream infections have been shown to be ten times more frequent in cirrhotic patients and are associated with poor outcomes including acute on chronic liver failure, acute kidney injury, encephalopathy and mortality [3–12]. The majority of infecting bacteria found in patients with cirrhosis are Gram-negative; however, there has been an increase in the prevalence of Gram-positive bacteria with concerns for enterococcal infection [10, 13]. Treatment options in patients with liver disease are challenging for several reasons. First, the pharmacokinetics of antibiotics change with liver disease due to several physiological abnormalities such as hypoalbuminemia, altered liver and renal functions [8, 14]. Second, previous studies have shown a possible association between cirrhosis, colonization and infection by antimicrobial-resistant bacteria as well as higher rates of enterococcal infections [7, 10, 11, 15, 16]. Guidelines for empirical treatment of hospital-acquired infections in cirrhotic patients vary considerably across countries, with third generation cephalosporins, piperacillin-tazobactam or carbapenem being recommended according to local antibiotic resistance data [17, 18]. The 2018 European association for the study of the Liver (EASL) guidelines suggest considering Gram-positive coverage in cases of hospital-acquired infections [17, 18]. The question arises whether extended coverage for Gram-positive and resistant Gram-negative bacteria should be recommended as a first-line treatment in the critically ill cirrhotic patients.

Epidemiological knowledge about the microorganisms responsible for HABSI in cirrhotic patients, their sources and the patient’s outcome in the ICU are scarce and no large comparison of the characteristics of these patients with a non-cirrhotic group has previously been performed [4, 8–11, 19]. The objectives of this study were to describe the differences in the epidemiology of HABSI, with particular attention to *E. faecium* infections, between cirrhotic and non-cirrhotic patients in terms of patients’ characteristics, source of infection, microorganism distribution and mortality using a large multicontinental database.

## Methods

### Setting

The Eurobact-2 study was a prospective observational international cohort study conducted between August 2019 and June 2021 [2, 20].

A total of 333 centers participated in the study which was registered in ClinicalTrials.org (NCT03937245). The study was reported in accordance with the STrengthening the Reporting of OBservational studies in Epidemiology (STROBE) guidelines [21].

Details regarding the methodology can be found in the previously reported Eurobact-2 study [2]. The study was approved by the ethics Committee of the Royal Brisbane & Women’s Hospital Human Research (LNR/2019/QRBW/48,376). Each study site then obtained ethical and governance approvals according to the local regulations in place [2].

### Intensive care unit and patient selection

Of the 333-participating centers of the Eurobact-2 study, we included only the centers that recruited HABSIs both in patients with and without cirrhosis for this analysis. Patients aged ≥ 18 years with a first episode of HABSI treated in the ICU were prospectively included. A HABSI was defined as a positive blood culture collected at least 48 h after hospital admission. Patients whose blood cultures were collected in the ICU (i.e., HABSI acquired in the ICU) and patients transferred to the ICU for treatment of HABSI were included. Only the first episode of HABSI was included. The presence of cirrhosis was determined based on the presence of mild to severe cirrhosis as indicated in the Charlson Comorbidity Index [22].

Blood cultures with possible skin contaminants (e.g., coagulase-negative staphylococci, *Corynebacterium* species) were carefully reviewed and included only if at least two blood cultures with the same antimicrobial susceptibility pattern were observed or if there was strong clinical suspicions that the blood culture was not a contaminant [2, 20]. Community-acquired bloodstream infections were excluded.

### Data collection

A center form was collected which described the ICU types and functioning. For each patient, data on ICU admission and on the day of the HABSI were collected. Further information on definitions is illustrated in the electronic supplementary material (ESM). Patients were followed for up to 28 days or until hospital discharge, or death.

### Statistical analysis

First, a descriptive analysis of patients’ characteristics on admission according to the presence of cirrhosis was performed. Continuous variables were presented as median with interquartile range (IQR) and categorical variables as number of patients (n) and percentage (%). Chi-square or Fisher tests were used to detect differences in categorical variables as appropriate and Wilcoxon rank sum test in continuous variables. Source of infection and microorganisms’ distribution were described in the same way.

Second, a descriptive analysis of the distribution of HABSI microorganisms according to the presence of cirrhosis was done. A multivariable mixed logistic regression was performed to investigate the association between *E. faecium* and cirrhosis with an adjustment for well-known risk factors for *E. faecium* infections (i.e., reason for ICU admission, source of infection, acquisition of the BSI in the ICU, use of antibiotics in the previous 7 days and delay between hospital admission and time of the BSI) [23, 24]. A random effect for the variable center was included. Results of the mixed logistic regression are expressed as odds ratios (OR).

Third, we investigated the association between cirrhosis and 28-day mortality using a graphical representation with Kaplan–Meier curves (with log-rank test). We analyzed the whole population and specifically the subgroup of patient with an *E. faecium* HABSI. The proportional hazard assumption was graphically assessed. Finally, we investigated the association between cirrhosis and mortality with a multivariable frailty Cox model with a random effect for center and adjusted for previously identified mortality risk factors (i.e., difficult-to-treat gram negative bacteria, absence of the consultation by a clinical pharmacist, source control, SAPS II on ICU admission) [2]. Moreover, a further adjustment to COVID-19 status was added since HABSI critically ill patients infected with COVID-19 showed higher mortality [20]. Results of the Cox regression analysis are expressed as hazard ratios (HR). Due to the very low number of missing data, a complete case analysis was performed.

Two-tailed *p*-values ≤ 0.05 were considered statistically significant. All statistical analyses are conducted using STATA version 16.1 (Stata Corp., College Station, TX, USA, 2007) and R (Version 3.5.3).

## Results

### Centers

Among the 333 centers recruited in the Eurobact-2 study, 232 centers were excluded as they did not include cirrhotic patients (Supplementary Figs. 1 and 2). Among the included centers, 75 were mixed ICUs (medical and surgical), 16 were medical ICUs and 9 were surgical ICUs. A mean of 1.6 (SD 0.9) cirrhotic patients per center were recruited. In the 101 selected centers 1059 patients were included, 899 of them were non-cirrhotic patients and 160 were cirrhotic. In those patients 1196 microorganisms were identified. Further details regarding centers are shown in the Supplementary Table 1.

### Patients’ characteristics

Patients characteristics on ICU admission and their outcomes according to the presence of cirrhosis are presented in Table 1. Patients median age was 64 years (IQR 53–73) and 62.5% (662/1059) were men. The primary causes for ICU admissions were septic shock (23.9%, 253/1059) and respiratory failure (21.2%, 225/1059). Cirrhotic patients were younger with a median age of 60 years (IQR 50.5–67) compared to 65 years (IQR 54–73, *p* < 0.01) for non-cirrhotic patients. Their comorbidities differed significantly with cirrhotic patients presenting more often with renal disease (21.3% (34/160) vs. 16% (144/899)) and non-cirrhotic with neurological comorbidities (8.1% (13/160) vs. 14.7% (132/899)), diabetes (21.9% (35/160) vs. 27% (243/899)) and malignancy (20% (32/160) vs. 26.3% (236/899), *p* < 0.01). On ICU admission, cirrhotic patients were more frequently admitted for an abdominal disease (15% (24/160) vs. 3.3% (30/899)) and for sepsis (32.5% (52/160) vs. 22.4% (201/899)) compared to non-cirrhotic patients (*p* < 0.01). The 28-day mortality was higher in cirrhotic patients (45% (72/160) vs. 36.4% (327/899), *p* = 0.04).

**Table 1:** Patients’ characteristics on admission and outcomes

*N* = 1059	Non-cirrhotic patients *n* = 899	Cirrhotic patients *n* = 160	*p* value
Age (years), median (IQR)	65 (54–73)	60 (50.5–67)	< 0.01
Gender, male, n(%)	551 (61.3%)	111 (69.4%)	0.052
Comorbidities, n (%): -Respiratory -Cardiovascular -Neurological -Diabetes -Renal insufficiency -Malignancy	147 (16.4%) 205 (22.8%) 132 (14.7%) 243 (27%) 144 (16%) 236 (26.3%)	25 (15.6%) 31 (19.4%) 13 (8.1%) 35 (21.9%) 34 (21.3%) 32 (20%)	< 0.01
ICU admission origin, n (%): -other hospital -emergency -OR -hospital ward -Intermediate care unit -other	133 (14.8%) 220 (24.5%) 111 (12.4%) 399 (44.4%) 22 (2.5%) 14 (1.6%)	15 (9.4%) 27 (16.9%) 23 (14.4%) 88 (55%) 6 (3.4%) 1 (0.6%)	0.04
Type of admission, n (%): -Medical -Surgical elective -Surgical emergency	671 (74.6%) 67 (7.5%) 161 (17.9%)	121 (75.6%) 10 (6.3%) 29 (18.1%)	0.9
Time between hospital and ICU admission (days), median (IQR)	4 (1–13)	5 (2–17)	0.4
Diagnosis on ICU admission, n (%) : -Cardiovascular disease -Respiratory disease -Neurological disease -Abdominal disease -Renal failure -Sepsis -Post-surgery -Others * -COVID-19	86 (9.6%) 200 (22.3%) 95 (10.6%) 30 (3.3%) 19 (2.1%) 201 (22.4%) 102 (11.4%) 52 (5.8%) 114 (12.7%)	10 (6.3%) 25 (15.6%) 14 (8.8%) 24 (15%) 2 (1.3%) 52 (32.5%) 15 (9.4%) 8 (5%) 10 (6.3%)	< 0.01
SAPS II on ICU admission, median (IQR)	46 (37–58)	48 (36.5–61)	0.1
Lactate (mmol/l) on ICU admission, median (IQR)	2.2 (1.4–3.9)	3 (1.7–5.1)	< 0.01
Bilirubin (µmol/L) on ICU admission, median (IQR)	1.8 (0.7–10.1)	7.5 (1.5–35)	< 0.01
WBC (x10^9^/L) on ICU admission, median (IQR)	14.3 (9.4–22.4)	14.5 (8.9–22.5)	0.7
CRP (mg/l) on ICU admission, median (IQR)	117.6 (33.5–215)	70 (23.2-155.6)	< 0.01
Need for renal replacement therapy during ICU stay, n (%)	126 (14%)	27 (16.9%)	0.3
Ventilation needs during ICU stay, n(%): -Mechanical ventilation -Non-invasive ventilation -High flow -low flow or nothing	546 (60.8%) 75 (8.4%) 75 (8.4%) 201 (22.4%)	92 (57.5%) 21 (13.1%) 10 (6.3%) 37 (23.1%)	0.2
Known MDRO before admission, n(%)	173 (19.3%)	28 (17.5%)	0.6
Antibiotics in the previous 7 days, n(%)	661 (73.5%)	119 (74.4%)	0.8
Days under mechanical ventilation, median (IQR)	6 (1–18)	6.5 (1–13)	0.4
ICU length of stay (days), median (IQR)	16 (7–27)	13.5 (6–30)	0.5
Death at day 28, n(%)	327 (36.4%)	72 (45%)	0.04

Results reported as *n* (%) for categorical variables and median (IQR) for continuous variables

ICU intensive care unit; OR operating room; WBC white blood cells; CRP C-reactive protein; MDRO multi-drug resistant organisms

*: multiple trauma with no traumatic brain injury, metabolic disturbances, drug overdose, diabetic ketoacidosis, anaphylaxis

### Distribution of microorganisms, antimicrobial resistance and antibiotics used before HABSI between cirrhotic and non-cirrhotic patients

**Fig. 1:**
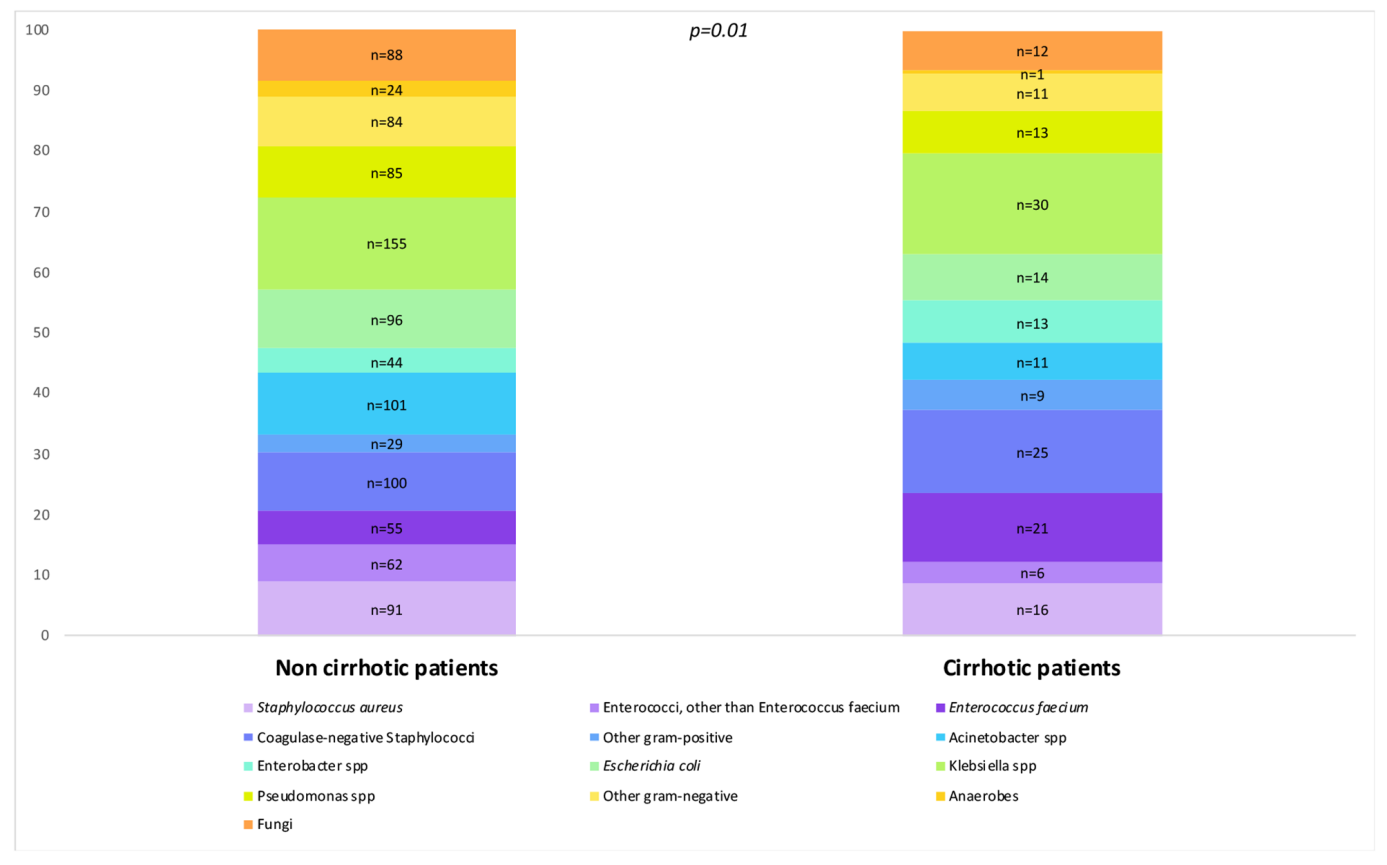
The distribution of microorganisms between cirrhotic and non-cirrhotic patients was compared, with the results reported as the number of each microorganism in the two different patient groups. The Fisher’s exact test was used for the comparison, revealing a significant difference in microorganism distribution between cirrhotic and non-cirrhotic patients (*p* = 0.01)

Of the 1196 microorganisms identified, 42.3% (77/182) of HABSIs in cirrhotic patients were Gram-positive bacteria compared to 33.2% (337/1014) in non-cirrhotic patients (*p* = 0.02) (Supplementary Table 2). Fig. 1 describes the distribution of microorganisms between cirrhotic and non-cirrhotic patients. *E. faecium* HABSI was found more often in cirrhotic patients (11.5% (21/182) vs. 4.5% (55/1014), *p* < 0.01). HABSI in cirrhotic patients were most frequently caused by *Klebsiella* spp (16.5% (30/182)), coagulase-negative Staphylococci (13.7% (25/182)) and *Enterococcus faecium* (11.5% (21/182)). HABSI in non-cirrhotic patients were more commonly caused by *Klebsiella* spp (15.3% (155/1014)), *Acinetobacter* spp (10% (101/1014)) and coagulase-negative Staphylococci (9.9% (100/1014)). No difference regarding antibiotic resistance and antibiotic exposure prior to HABSI was highlighted between the two groups (Supplementary Tables 3 and 4.). Interestingly, proportions of *Klebsiella* spp and *E. coli* resistant to third generation cephalosporins were 50% in cirrhotic patients and 55.8% in non-cirrhotic patients (*p* = 0.5).

### Characteristics of HABSI and sources of infection

HABSIs were more frequently acquired in the ICU than on the ward in both groups (Table 2). Compared to non-cirrhotic patients, cirrhotic patients developed HABSI on the ward more often (31.9% (51/160) vs. 23.9% (215/899), *p* = 0.03). The most common source of HABSI in cirrhotic patient was abdominal (35.6% (57/160)) whereas it was pulmonary (18.9% (207/899)) for non-cirrhotic patients (*p* < 0.01). Patients’ characteristics on HABSI day are illustrated in the Supplementary Table 5.

**Table 2:** Characteristics of HABSI and sources of infection

*N* = 1059	Non-cirrhotic patients *n* = 899	Cirrhotic patients *n* = 160	*p* value
Time to HABSI (days), median (IQR)	14 (8–26)	15 (8–25)	0.6
Site of acquisition, n(%) -Ward -ICU	215 (23.9%) 684 (75.1%)	51 (31.9%) 109 (68.1%)	0.03
Supposed source of the HABSI, n (%) -primary -catheter related -pulmonary -abdominal -urinary tract -cutaneous or soft tissue -others*	110 (12.2%) 170 (18.9%) 207 (23%) 169 (18.8%) 105 (11.7%) 63 (7%) 75 (8.3%)	19 (11.9%) 24 (15%) 23 (14.4%) 57 (35.6%) 16 (10%) 10 (6.3%) 11 6.9%)	< 0.01
Source control, n (%) -not required -required and complete -required but unsuccessful	381 (42.4%) 426 (47.4%) 92 (10.2%)	75 (46.9%) 71 (44.4%) 14 (8.8%)	0.6
Appropriate antibiotic in the first 24 h, n (%)	459 (51.1%)	78 (48.7%)	0.6

Results reported as *n* (%) for categorical variables and median (IQR) for continuous variables

*mediastinitis, endocarditis, joints, central nervous system

*HABSI* hospital-acquired bloodstream infection; ICU intensive care unit

### Association between *E. faecium* and cirrhosis

Using a multivariable mixed logistic regression model and after adjustment for well-known *E. faecium* risk factors, cirrhosis was associated with a higher risk of *E. faecium* HABSI (OR 2.5, 95% CI 1.3–4.5, *p* < 0.01, Table 3). Description of patients with *E. faecium* HABSI is shown in the Supplementary Table 6. Importantly, 84% of *E. faecium* HABSI were acquired in the ICU.

**Table 3:** Multivariable logistic mixed model for the association between *E. faecium* and cirrhosis

*n* = 1059	E. faecium BSI, Odds ratio (95% CI)	*p* value
**Cirrhosis**	**2.5 (1.3–4.5)**	**< 0.01**
Reason for ICU admission:		
-Cardio-vascular disease	Ref.	
-Cardio-vascular disease	1.5 (0.5–4.4)	0.4
-Neurological disease	0.3 (0.06–1.8)	0.2
-Abdominal disease	1.5 (0.5–5.2)	0.5
-Post-surgical treatment	0.9 (0.3–3.1)	0.9
-Renal failure	1.2 (0.1–11)	0.9
-Septic Shock	0.7 (0.2-2)	0.5
-COVID-19	3.2 (1.1–10)	0.04
-other*	0.7 (0.1–3.1)	0.6
Source of infection:		
-Primary	Ref.	
-Intravascular catheter related	0.6 (0.3–1.5)	0.3
-Pulmonary	0.2 (0.1–0.6)	< 0.01
-Abdominal	0.4 (0.1–1.3)	0.5
-Urinary	0.4 (0.1–1.3)	0.1
-Skin	0.9 (0.3-3)	0.9
-Other†	0.6 (0.2-2)	0.4
BSI acquired before ICU admission	0.6 (0.3–1.3)	0.2
Antibiotics in the last 7 days	1.9 (0.9–3.9)	0.056
Delay (days) between hospital admission and BSI	0.9 (0.9–1.1)	0.7

Results are expressed as odds ratios and 95% confidence interval (95% CI). A random effect for center was included

*HABSI* hospital-acquired bloodstream infection

*: multiple trauma with no traumatic brain injury, metabolic disturbances, drug overdose, diabetic ketoacidosis, anaphylaxis

†: mediastinitis, endocarditis, joints, central nervous system

### Association between cirrhosis and mortality

**Fig. 2:**
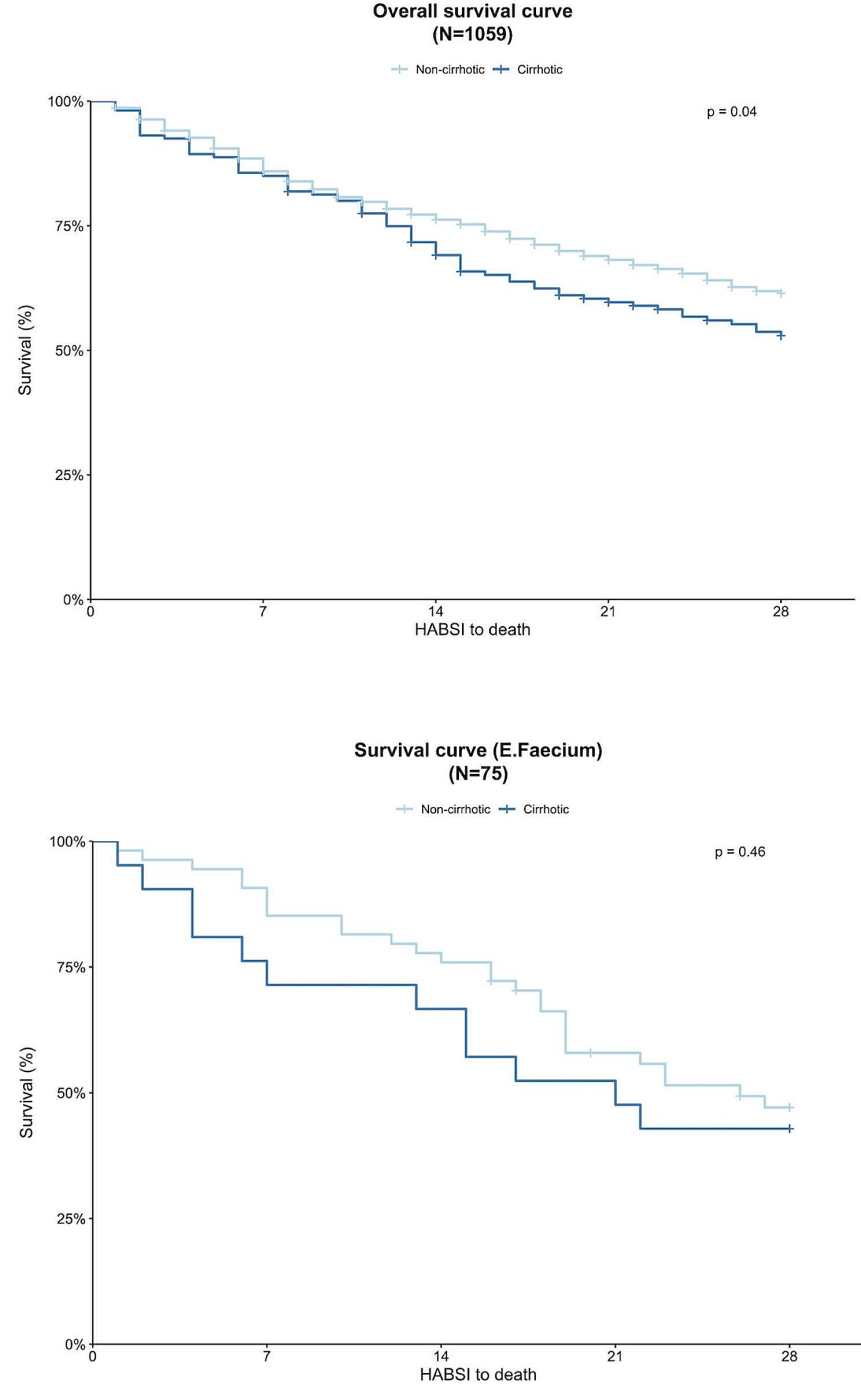
Kaplan-Meier survival curves were used to analyze survival until Day 28 for all HABSIs and for *Enterococcus faecium* HABSI in cirrhotic and non-cirrhotic patients. Statistical analysis of the Kaplan-Meier curve (log-rank test) showed a lower probability of survival in cirrhotic patients with HABSI (*p* = 0.04) and no significant difference in cirrhotic patients with *E. faecium* HABSI compared to non-cirrhotic patients (*p* = 0.46)

Mortality on day 28 was higher in cirrhotic patients compared to non-cirrhotic patients (*p* = 0.04) (Fig. 2). The proportionality assumption was respected. A multivariable frailty Cox model showed an increased risk of death in HABSI patients known for cirrhosis (HR 1.3, 95% CI 1.01–1.7, *p* = 0.045, Supplementary Table 7). Cirrhotic patients with *E. faecium* HABSI tended to have an even higher 28-day mortality; however, this result was non-significant.

## Discussion

In this analysis of a prospective multicontinental cohort of patients with HABSI, we found that patients with liver cirrhosis had higher mortality than those without cirrhosis. HABSI in cirrhotic patients were more frequently due to Gram-positive bacteria, especially *E. faecium*, than in non-cirrhotic patients. No difference regarding antimicrobial resistance was observed between the two groups.

Previous studies showed similar rates of Gram-positive (26.3–49%) and Gram-negative (48–57.2%) HABSI in cirrhotic patients [10, 15]. These studies found that 2 to 9% of HABSI were due to *E. faecium*. The differences and limitations of these previous studies are that (1) they were monocentric or included only a limited number of centers, (2) they did not focus on ICU patients and, (3) they did not perform a comparison between cirrhotic and non-cirrhotic patients. Using a large multicontinental database that prospectively included patients, we observed that the rate of Gram-positive bacteremia was higher in critically ill cirrhotic patients than in non-cirrhotic patients, with *E. faecium* much more prevalent among cirrhotic patients. There are several hypotheses for this finding. First, the source of the bacteremia in cirrhotic patients was more often abdominal and enterococci are known to colonize the intestinal tract. Second, cirrhotic patients are exposed to repeated prophylactic and therapeutic antibiotic treatments. Indeed, these patients frequently receive antibiotics because they are more prone to infections and especially to spontaneous bacterial peritonitis [6]. Interestingly, we did not observe differences in antibiotics administered 7 days before HABSI. However, since we had no access to data on antibiotic therapies in the months before HABSI, it is conceivable that cirrhotic patients were more frequently exposed to antimicrobial substances which might have impacted the epidemiology of their HABSI. Lastly, our population included only HABSI and this setting is characterized by higher prevalence of *E. faecium*, whereas E. *faecalis* tends to occur more frequently in the community-acquired setting [25].

Our results have clinical implications. The high rate of *E. faecium* HABSI found in cirrhotic patients may challenge empirical antibiotic therapies that should be proposed in this particular setting. A study showed that clinicians felt that in cases of severe sepsis, 90% of all probable microorganisms should be covered by the empirical antibiotics chosen [26]. As *E. faecium* accounts for more than 10% of HABSI observed in these critically ill cirrhotic patients, empiric coverage with glycopeptides, daptomycin or oxazolidinones should be considered. This coverage seems particularly important if the suspected source is abdominal: a recent multicentric study found that intra-abdominal infections in critically ill patients growing *Enterococcus* were associated with a higher mortality when there was no empirical coverage of *E. faecium* [27].

Regarding other specific resistances to antibiotics in the context of cirrhosis, some previous studies have reported higher rates of drug-resistant organisms such as extended spectrum beta-lactamase (ESBL) or methicillin-resistant *Staphylococcus aureus* (MRSA), depending highly on the local ecology [7, 10, 11]. However, our study did not show any difference in antimicrobial resistance between patients with and without cirrhosis. Therefore, cirrhosis per se does not seem to influence the risk of resistance relative to other patients in the ICU with HABSI, but it mostly impacts the distribution of strains causing HABSI. Interestingly, the proportion of *Klebsiella* spp and *E coli* spp resistant to third-generation cephalosporins represented almost 50% of all HABSI in the Eurobact-2 cohort without differences between cirrhotic and non-cirrhotic patients. This finding seems to be associated with the local ecology; therefore, no firm conclusion on empirical antibiotic therapy for Gram-negative microorganisms for cirrhotic patients can be provided.

Our study revealed that there was no significant difference in the incidence of fungal HABSI between cirrhotic and non-cirrhotic patients. This finding is noteworthy as these infections are often concerning in cirrhotic patients as previous studies highlighted more invasive fungal infections in cirrhotic patients compared to non-cirrhotic patients, especially in an ICU setting [28, 29]. However, the results of our study suggest that cirrhotic patients may not be at a higher risk for fungal infections, which has important implications for clinical management strategies.

Our study highlights that critically ill cirrhotic patients with HABSI were associated with poorer outcomes, with a mortality rate reaching 45%, which was significantly higher than non-cirrhotic patients. This has already been highlighted for nosocomial infections in cirrhotic patients [30]. Active measures to prevent nosocomial infections, including HABSI, should be thoroughly applied to these vulnerable patients. This can be achieved through excellent infection prevention and control measures targeting the most important healthcare-associated infections, reducing the unnecessary use of proton pump inhibitors, avoiding the placement of intravascular and urinary catheters and limiting their duration [30, 31].

Our study has several limitations. First, ICUs in high income countries and European ICUs were overrepresented with three countries (France, UK and Turkey) recruiting 30% of patients, thus potentially limiting the generalizability of our results [2]. However, each continent was represented and included patients in our study. Second, pathogen identification and antimicrobial susceptibility testing was dependent on each center’s laboratory, which limits the standardization of microbiological results [2]. Third, the causes and severity of cirrhosis could not be assessed, which could have influenced the outcome [13]. Fourth, data collection was performed by individual investigators in each ICU without on-site monitoring. This limitation was controlled by providing online checks through the electronic case report file and by monitoring data quality and coherence of the data for each case-report form [2]. Lastly, this is a secondary analysis of the Eurobact-II database, with the inherent risk of bias that such analyses may carry.

In summary, critically ill cirrhotic patients with HABSI have a higher mortality than those without cirrhosis. They present a specific microbiology, with more Gram-positive bacteria and especially more *E. faecium* bacteremia than non-cirrhotic patients. In these particular patients, empirical coverage of *E. faecium* should be considered. This should be confirmed by interventional studies specifically designed to determine the efficacy and safety of such antibiotic treatments in this high-risk population.

### Electronic supplementary material

Below is the link to the electronic supplementary material.

Supplementary Material 1

Supplementary Material 2

## Data Availability

The datasets used and/or analyzed during the current study are available from the corresponding author on reasonable request.

## Abbreviations

HABSI: Hospital-acquired bloodstream infections
ICU: intensive care unit
SAPSII: simplified acute physiology score II
SOFA: sequential organ failure assessment
OR: operating room
WBC: white blood cells
CRP: C-reactive protein
CVVHDF: continuous venovenous hemodiafiltration
SLEDD: Sustained low-efficiency daily dialysis
MDRO: multi-drug resistant organisms
