# Supplementary material for: Hospital-acquired bloodstream infections in critically ill cirrhotic patients: a post-hoc analysis of the EUROBACT-2 international cohort study
Source: Ann Intensive Care. 2024 May 2;14:70. doi: 10.1186/s13613-024-01299-x (PMC11065852; doi:10.1186/s13613-024-01299-x)
Supplement: Supplementary file 1 — Supplementary Material 1 [file 13613_2024_1299_MOESM1_ESM.docx]

**Online Data Supplement**

**Hospital-Acquired Bloodstream Infections in Critically Ill Cirrhotic Patients: a Post-hoc Analysis of the EUROBACT-2 International Cohort Study**

**Authors**

Hannah Wozniak^1,2^, Alexis Tabah^3,4,5,6^, François Barbier^7^, Stéphane Ruckly^8,9^, Ambre Loiodice^9^, Murat Akova^10^, Marc Leone^11^, Andrew Conway Morris^12,13,14^, Matteo Bassetti^15^, Kostoula Arvaniti^16^, Ricard Ferrer^17^, Liesbet de Bus^18,19^, Jose Artur Paiva^20,21^, Hendrik Bracht^22^, Adam Mikstacki^23,24^, Adel Alsisi^25,26^, Liana Valeanu^27^, Josef Prazak^28^, Jean-François Timsit^29,30^, Niccolò Buetti^29,31^ on behalf of the EUROBACT‐2 Study Group, ESICM, ESCMID ESGCIP and the OUTCOMEREA Network

^1^ Interdepartmental Division of Critical Care Medicine, University of Toronto, Toronto, Canada
^2^ Intensive Care Unit, Geneva University Hospitals, Geneva, Switzerland

^3^ Intensive Care Unit, Redcliffe Hospital, Brisbane, Australia.

^4^ Queensland Critical Care Research Network (QCCRN), Brisbane, QLD, Australia

^5^ Queensland University of Technology, Brisbane, QLD, Australia.

^6^ Faculty of Medicine, The University of Queensland, Brisbane, QLD, Australia.

^7^ Service de Médecine Intensive‐Réanimation, Centre Hospitalier Régional d’Orléans

^8^ Université de Paris, INSERM, IAME UMR 1137, 75018 Paris, France.

^9^ ICUREsearch, Biometry, 38600 Fontaine, France.

^10^ Department of Infectious Diseases, Hacettepe University School of Medicine, Ankara, Turkey

^11^ Department of Anesthesiology and Intensive Care Unit, Hospital Nord, Aix Marseille University, Assistance Publique Hôpitaux Universitaires de Marseille, Marseille, France.

^12^ Division of Anaesthesia, Department of Medicine, University of Cambridge, Addenbrooke’s Hospital, Hills Road, Cambridge CB2 0QQ, UK.

^13^ Division of Immunology, Department of Pathology, University of Cambridge, Tennis Court Road, Cambridge Cb2 1QP, UK

^14^ JVF Intensive Care Unit, Addenbrooke’s Hospital, Cambridge, Hills Road, Cambridge CB2 0QQ, UK

^15^ Infectious Diseases Clinic, Department of Health Sciences, University of Genoa and Ospedale Policlinico San Martino, Genoa, Italy.

^16^ Intensive Care Unit, Papageorgiou University Affiliated Hospital, Thessaloníki, Greece

^17^ Intensive Care Department, SODIR‐VHIR Research Group, Vall d’Hebron University Hospital, Barcelona, Spain.

^18^ Department of Critical Care Medicine, Ghent University Hospital, Ghent, Belgium

^19^ Department of Internal Medicine and Paediatrics, Faculty of Medicine and Health Sciences, Ghent University, Ghent, Belgium

^20^ Intensive Care Medicine Department, Centro Hospitalar Universitário São João (CHUSJ), Porto, Portugal.

^21^ Department of Medicine, Faculty of Medicine, University of Porto (FMUP), Porto, Portugal

^22^ Central Interdisciplinary Emergency Medicine, University Hospital Ulm, Ulm, Germany.

^23^ Faculty of Health Sciences, Poznan University of Medical Sciences, Poznan, Poland

^24^ Department of Anaesthesiology and Intensive Therapy, Regional Hospital in Poznan, Poznan, Poland.

^25^ ICU Department, Prime Hospital, Dubai, United Arab Emirates

^26^ Critical Care Department, Faculty of Medicine, Cairo University, Cairo, Egypt.

^27^ Cardiac Anesthesiology and Intensive Care Department I, Emergency Institute for Cardiovascular Diseases Prof. Dr. C. C. Iliescu, Bucharest, Romania.

^28^ Department of Intensive Care Medicine, Inselspital, Bern University Hospital, University of Bern, Bern, Switzerland

^29^ Université Paris‐ Cité, INSERM, IAME UMR 1137, 75018 Paris, France.

^30^ Medical and Infectious Diseases Intensive Care Unit, AP‐HP, Bichat‐Claude Bernard University Hospital

^31^ Infection Control Program and World Health Organization Collaborating Centre on Patient Safety, University Hospitals and Faculty of Medicine, University of Geneva, Geneva, Switzerland.

Table of contents

Protocol and Definitions…………………………………………………………...3

Figure S1..……………………………………………………………………….…4

Table S1……………………………………………………………………………5

Figure S2..………………………………………………………………………….6

Table S2……………………………………………………………………………6

Table S3……………………………………………………………………………7

Table S4……………………………………………………………………………7

Table S5……………………………………………………………………………7

Table S6……………………………………………………………………………9

Table S7……………………………………………………………………………9

**Protocol and Definitions**

**Data collection**

Data collection was performed by individual investigators in each ICU without on-site monitoring. A dual verification process that included a set of consistency routines that automatically checked all data plus a manual check of each case report was done by a group of experts for data quality, coherence and completeness. Any issues or inconsistencies were referred back to the center investigator and rechecked until the case was satisfactorily completed [1, 2].

Reference

1. Buetti N, Tabah A, Loiodice A, Ruckly S, Aslan AT (2022) Different epidemiology of bloodstream infections in COVID ‑ 19 compared to non ‑ COVID ‑ 19 critically ill patients : a descriptive analysis of the Eurobact II study. 1–12

2. Tabah A, Buetti N, Staiquly Q, Ruckly S, Akova M, Aslan AT, Leone M, Morris AC, Bassetti M, Arvaniti K (2023) Epidemiology and outcomes of hospital ‑ acquired bloodstream infections in intensive care unit patients : the EUROBACT ‑ 2 international cohort study. https://doi.org/10.1007/s00134-022-06944-2

**Figure. S1. Study flowchart**

**
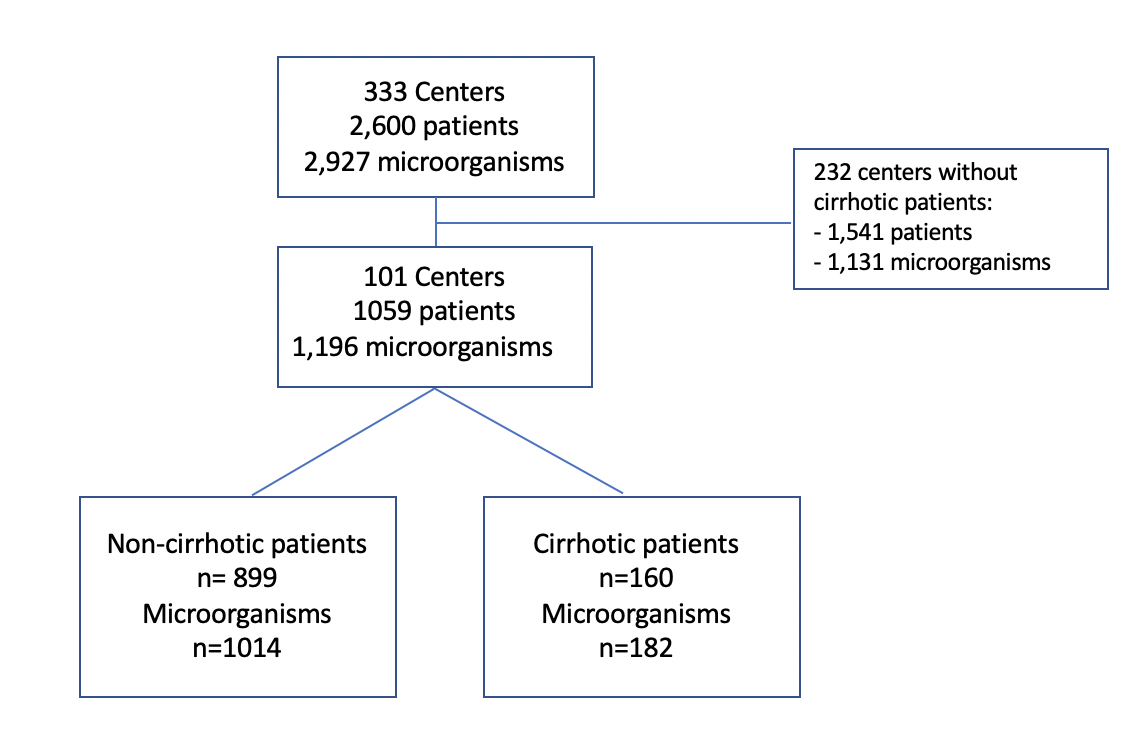
**

**Table S1. Centers and countries description**

|  |  | Centers, n=101 |
| --- | --- | --- |
| Country |  |  |
|  | UK | 10 (9.9%) |
|  | Turkey | 7 (6.9%) |
|  | Thailand | 1 (1%) |
|  | Taiwan | 2 (2%) |
|  | Switzerland | 2 (2%) |
|  | Spain | 5 (5%) |
|  | South Africa | 1 (1%) |
|  | Singapore | 2 (2%) |
|  | Russian Federation | 4 (4%) |
|  | Romania | 3 (3%) |
|  | Republic of Korea | 4 (4%) |
|  | Republic of Ireland | 1 (1%) |
|  | Portugal | 4 (4%) |
|  | Poland | 2 (2%) |
|  | Mexico | 2 (2%) |
|  | Kazakhstan | 1 (1%) |
|  | Japan | 1 (1%) |
|  | Italy | 4 (4%) |
|  | Israel | 1 (1%) |
|  | India | 1 (1%) |
|  | Hong Kong | 1 (1%) |
|  | Greece | 6 (5.9%) |
|  | Germany | 3 (3%) |
|  | France | 13 (12.9%) |
|  | Egypt | 4 (4%) |
|  | Croatia | 1 (1%) |
|  | China | 2 (2%) |
|  | Canada | 1 (1%) |
|  | Brunei | 1 (1%) |
|  | Bosnia and Herzegovina | 1 (1%) |
|  | Belgium | 4 (4%) |
|  | Bangladesh | 1 (1%) |
|  | Australia | 5 (5%) |
| Type of Hospital, n (%)* | Non-teaching | 14 (14%) |
|  | teaching | 86 (86%) |
| Type of ICU, n (%) | Mixed (medical-surgical) | 75 (75%) |
|  | Medical | 16 (16%) |
|  | Surgical | 9 (9%) |
| Structure of ICU, n (%)* | Closed ICU | 80 (80%) |
|  | Open ICU | 20 (20%) |
| Funding, n (%)* | Public | 84 (84%) |
|  | Private | 10 (10%) |
|  | Mixed | 6 (6%) |
| Number of patients per center, median (IQR) |  | 10 (6-11) |
| Number of cirrhotic patients per center, median (IQR) |  | 1 (1-2) |
| Consult of a clinical pharmacist on a regular basis* |  | 66 (66%) |

*one missing data

Results reported as *n* (%) for categorical variables and median (IQR) for continuous variables.

ICU intensive care unit

**Figure S2. Geographical distribution of the patients included**

**
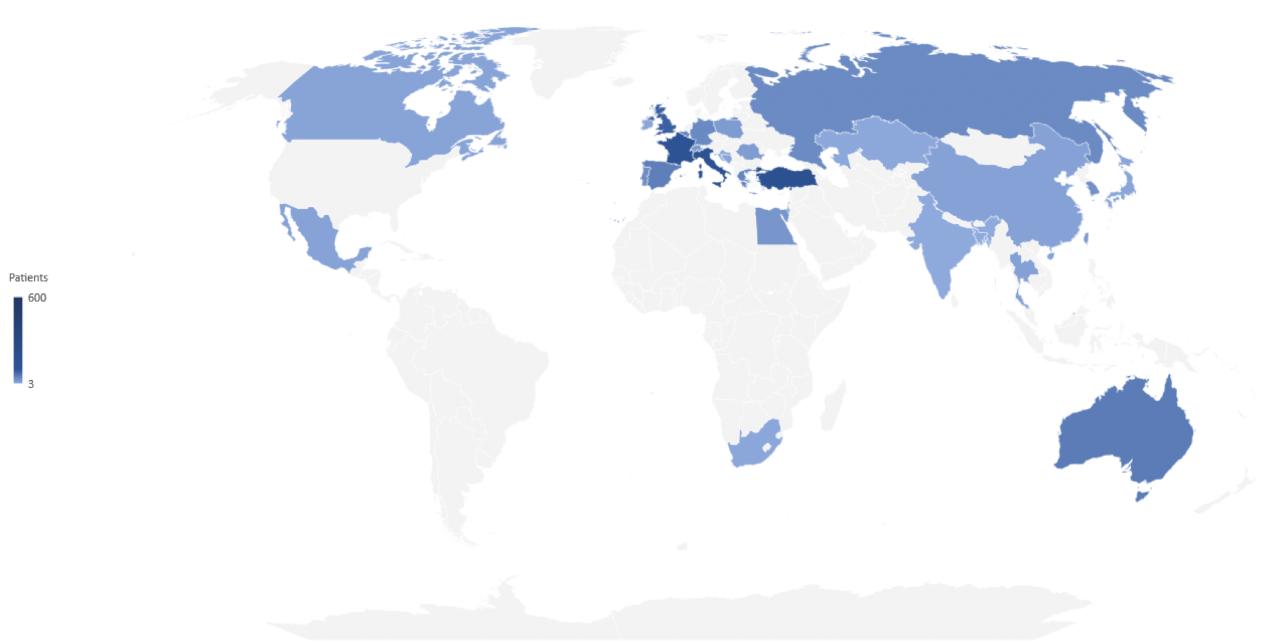
**

**Table S2. Distribution of microorganisms between cirrhotic and non-cirrhotic patients**

| Number of bacteriemia, n= 1196 | Non-cirrhotic patients  n= 1014 | Cirrhotic patients  n =182 | *p* value |
| --- | --- | --- | --- |
| Gram-positive, n (%) | 337 (33.2%) | 77 (42.3%) | 0.02 |
| *Staphylococcus aureus* | 91 (9%) | 16 (8.8%) | 0.9 |
| Coagulase-negative Staphylococci | 100 (9.9%) | 25 (13.7%) | 0.1 |
| Enterococci, other than *Enterococcus faecium* | 62 (6.1%) | 6 (3.3%) | 0.2 |
| *Enterococcus faecium* | 55 (5.4%) | 21 (11.5%) | <0.01 |
| Other Gram-positive bacteria | 29 (2.9%) | 9 (5%) | 0.1 |
| Gram-negative*,* n (%) | 565 (55.7%) | 92 (50.6%) | 0.2 |
| *Acinetobacter* spp | 101 (10%) | 11 (6%) | 0.095 |
| *Pseudomonas* spp | 85 (8.4%) | 13 (7.1%) | 0.6 |
| *Enterobacter* spp | 44 (4.3%) | 13 (7.1%) | 0.1 |
| *Escherichia coli* | 96 (9.5%) | 14 (7.7%) | 0.4 |
| *Klebsiella spp* | 155 (15.3%) | 30 (16.5%) | 0.7 |
| Other Gram-negative bacteria | 84 (8.3%) | 11 (6%) | 0.3 |
| Anaerobes*,* n (%) | 24 (2.4%) | 1 (0.6%) | 0.1 |
| Fungi*,* n (%) | 88 (8.7%) | 12 (6.6%) | 0.4 |

Results reported as *n* (%).

**Table S3. Antimicrobial resistance differences among selected microorganism groups**

| n= 1059 | Non-cirrhotic patients  n= 1014 | Cirrhotic patients  n=182 | *p* value |
| --- | --- | --- | --- |
| Resistant patterns*,* n (%)  -Pan-drug resistant among Gram-negative bacteria  -VRE  -MRSA  -MRSE  -*Klebsiella* spp and *E. Coli* resistant to third generation cephalosporin*  -carbapenem resistant  -resistant Gram negative (DTR)  -resistant Gram-positive microorganisms | -8/533 (1.5%)  -12/117 (10.3%)  -36/90 (40%)  -79/96 (82.3%)  -125/224 (55.8%)  -185/899 (20.6%)  -93/533(17.5%)  -127/316 (40.2% | -1/86 (1.2%)  -5/27 (18.5%)  -3/16 (18.7%)  -16/22 (72.7%)  -20/40 (50%)  -31/160 (19.4%)  -13/86 (15.1%)  -24/69 (34.8%) | 0.8  0.3  0.1  0.3  0.5  0.7  0.4  0.4 |

Results reported as *n* (%).

VRE Vancomycin-resistant enterococci; MRSA methicillin-resistant *Staphylococcus aureus*; MRSE Methicillin-resistant *Staphylococcus epidermidis*; DTR difficult to treat resistance; ESBL extended spectrum beta-lactamase

*31 missing data

**Table S4. Antibiotics administered in the 7 days before the HABSI**

| n= 780 | Non-cirrhotic patients  n= 661 | Cirrhotic patients  n= 119 | *p* value |
| --- | --- | --- | --- |
| Ceftriaxone*,* n (%)  Trimethoprim/sulfamethoxazole*,* n (%) Piperacillin-tazobactam*,* n (%)  Carbapenems*,* n (%)  Quinolones*,* n (%)  Others*,* n (%) | 49 (7.4%)  12 (1.8%)  142 (21.5%)  35 (5.3%)  107 (16.2%)  316 (47.8%) | 11 (9.2%)  3 (2.5%)  22 (18.5%)  4 (3.4%)  23 (19.3%)  56 (47.1%) | 0.9 |

Results reported as *n* (%).

HABSI hospital acquired bloodstream infection

**Table S5. Patient’s characteristic on HABSI day**

| n= 1059 | Non-cirrhotic patients  n= 899 | Cirrhotic patients  n =160 | *p* value |
| --- | --- | --- | --- |
| Patient presents with septic shock, n(%) | 263 (29.3%) | 62 (38.8%) | 0.02 |
| Tmax (°C) on HABSI day, median (IQR) | 38 (37.2-38.6) | 37.7 (37-38.5) | 0.052 |
| SOFA on HABSI day, median (IQR) | 7 (5-11) | 10 (7-13) | <0.01 |
| Lactate (mmol/L)on HABSI day, median (IQR) | 1.9 (1.3-3) | 2.4 (1.5-4.6) | <0.01 |
| Bilirubin (μmol/L) on HABSI day, median (IQR) | 2 (0.7-10) | 7.5 (1.6-34.5) | <0.01 |
| WBC (x10^9^/L)on HABSI day, median (IQR) | 14.6 (10.1-23.9) | 15.4 (9.7-25.5) | 0.9 |
| CRP (mg/L) on HABSI day, median (IQR) | 139.8 (59-224) | 78 (32.9-150) | <0.01 |

Results reported as *n* (%) for categorical variables and median (IQR) for continuous variables.

BSI bloodstream infection; Tmax maximal body temperature; WBC white blood cells; CRP C-reactive protein

**Table S6. *Enterococcus faecium* HABSI in cirrhotic patients**

| N= 75 | Non-cirrhotic patients  n =54 | cirrhotic patients  n =21 | *p* value |
| --- | --- | --- | --- |
| Age, median (IQR) | 67 (59-74) | 55 (45-64) | <0.01 |
| Gender, male, n(%) | 33 (61.1%) | 14 (66.7%) | 0.6 |
| Comorbidities, n (%):  -Respiratory  -Cardio-vascular  -Neurological  -Diabetes  -Renal insufficiency  -malignancy | 3 (5.6%)  6 (11.1%)  3 (5.6%)  10 (18.5%)  8 (14.8%)  4 (7.4%) | 0 (0%)  0 (0%)  0 (0%)  1 (4.8%)  5 (23.8%)  0 (0%) | 0.02 |
| ICU admission origin, n (%):  -other hospital  -emergency  -OR  -hospital ward  -Intermediate care unit  -other | 11 (20.4%)  12 (22.2%)  6 (11.1%)  24 (44.4%)  0 (0%)  1 (1.9%) | 1 (4.8%)  1 (4.8%)  6 (28.6%)  12 (57.1%)  1 (4.8%)  0 (0%) | 0.046 |
| Type of admission, n (%):  -Medical  -Surgical elective  -Surgical emergency | 40 (74.1%)  2 (3.7%)  12 (22.2%) | 15 (71.4%)  2 (9.5%)  4 (19.1%) | 0.6 |
| Time spent in the hospital before ICU admission, median (IQR) | 4.5 (1-17) | 5 (3-10) | 0.4 |
| Diagnosis on ICU admission, n (%) :  -Cardiovascular disease  -Respiratory disease  -Neurological disease  -Abdominal disease  -Renal failure  -Sepsis  -Post-surgery  -Others  -COVID-19 | 4 (7.4%)  12 (22.2%)  1 (1.9%)  3 (5.6%)  1 (1.9%)  11 (20.4%)  5 (9.3%)  2 (3.7%)  15 (27.8%) | 2 (9.5%)  3 (14.3%)  1 (4.8%)  6 (28.6%)  0 (0)  1 (4.8%)  5 (23.8%)  1 (4.8%)  2 (9.5%) | 0.06 |
| Site of acquisition of the HABSI, n(%)  -Ward  -ICU | 10 (18.5%)  44 (81.5%) | 2 (9.5%)  19 (90.5%) | 0.5 |
| SAPS II on ICU admission, median (IQR) | 46 (37-57) | 50 (47-67) | 0.1 |
| Source of HABSI n (%) :  -Primary  -Intravascular catheter related  -Pulmonary  -Abdominal  -Urinary  -Skin  -Other** | 10 (18.5%)  11 (20.4%)  6 (11.1%)  18 (33.3%)  3 (5.6%)  3 (5.6%)  3 (5.6%) | 4 (19.1%)  3 (14.3%)  1 (4.8%)  8 (38.1%)  2 (9.5%)  2 (9.5%)  1 (4.8%) | 0.9 |
| Need for CVVHDF/SLEDD during ICU stay, n (%) | 14 (25.9%) | 7 (33.3%) | 0.5 |
| Ventilation needs during ICU stay, n(%):  -ETT  -NIV  -High flow  -low flow or nothing | 31 (57.4%)  5 (9.3%)  8 (14.8%)  10 (18.5%) | 16 (76.2%)  3 (14.3%)  0 (0%)  2 (9.5%) | 0.2 |
| Known MDRO before admission, n(%) | 8 (14.8%) | 6 (28.6%) | 0.2 |
| Antibiotics in the previous 7d, n(%) | 43 (79.6%) | 19 (90.5%) | 0.3 |
| Days under mechanical ventilation, median (IQR) | 9 (1-16) | 7 (3-13) | 0.9 |
| ICU LOS, median (IQR) | 23.5 (9-28) | 19 (18-42) | 0.5 |
| Death at day 28, n(%) | 27 (50%) | 12 (57.1%) | 0.6 |

Results reported as *n* (%) for categorical variables and median (IQR) for continuous variables.

ICU intensive care unit; OR operating room; WBC white blood cells; CRP C-reactive protein; CVVHDF continuous venovenous hemodiafiltration; SLEDD Sustained low-efficiency daily dialysis; MDRO multi-drug resistant organisms

**Table S7. Multivariable frailty Cox model for the association cirrhosis and mortality**

| n=1057 | Mortality, HR (95% CI) | p |
| --- | --- | --- |
| **Cirrhosis**  Difficult-to-treat gram negative bacteria  No consultation by a clinical pharmacist  Source control:  -not required  -required and complete  -required but not achieved  SAPS II on ICU admission  Reason for ICU admission: COVID-19 | **1.3 (1.01-1.7)**  1.8 (1.3-2.4)  0.9 (0.7-1.3)  1  0.6 (0.5-0.8)  2 (1.5-2.7)  1.02 (1.01-1.03)  2.2 (1.6-3) | **0.045**  <0.01  0.9  <0.01  <0.01  <0.01  <0.01  <0.01 |

Results are expressed as hazard ratios (HR) and 95% confidence interval (95% CI). A random effect for center was included.

ICU intensive care unit.
