# Supplementary material for: Hospital-acquired bloodstream infections in critically ill cirrhotic patients: a post-hoc analysis of the EUROBACT-2 international cohort study
Source: Ann Intensive Care. 2024 May 2;14:70. doi: 10.1186/s13613-024-01299-x (PMC11065852; doi:10.1186/s13613-024-01299-x)
Supplement: Supplementary file 2 — Supplementary Material 2 [file 13613_2024_1299_MOESM2_ESM.docx]

**Answers to Reviewers – R2**

Dear Reviewers and Editors,

We would like to express our sincere gratitude for the insightful review and constructive comments provided for our manuscript.

We believe our study contributes significantly to the field of infectious complications in critically ill cirrhotic patients. Using large international prospectively collected data, we address a crucial knowledge gap by exploring the epidemiological distinctions between hospital-acquired bloodstream infections in cirrhotic and non-cirrhotic critically ill patients. Thanks to the Reviewers thoughtful comments, we have made substantial improvements to the manuscript.

Once again, we are grateful for this review process and for your suggestions.

Thank you for your time and consideration.

Sincerely,

Hannah Wozniak, MD

Niccolò Buetti, MD MSc PhD

On behalf of the Eurobact-2 study team

**Answers to Reviewer #1**

Reviewer #1 : The authors outline the putative association found between HABSI related to E faecium and prognosis among cirrhotic patients. But this has to be demonstrated. Whereas E faecium related
HABSI are more frequent among cirrhotic patient, no relation does existing regarding prognosis.
Moreover the more important rate of E faecium compared to E faecalis is not easy to understood in
the present study. It is hypothesized that antibiotic could selectively encourage the growth f E
faecium; but no available information reinforces this statement
Buetti reported an increased rate of E faecium among bactaeremia with duration of hospitalization
whereas the incidence of E faecalis bacteremia was table over time.
It would be of interest to have information as whether E faecium HABSI were more frequently
acquired in the ward or in ICU:
In Table 3 the multivariable logistic mixed model takes into account pulmonary source of infection,
which is extremely rare regarding enterococcus species and is therefore debatable.

Answer: We thank the Reviewer for this comment and suggestions.

1. Regarding the association between *E faecium* HABSI and Prognosis among Cirrhotic Patients

We appreciate the Reviewer's attention to the potential association between HABSI related to *E faecium* and prognosis among cirrhotic patients. Our study primarily aimed to provide epidemiological insights into HABSI in cirrhotic patients hospitalized in the ICU, as they represent a unique and fragile group. Our key findings indicate that critically ill cirrhotic patients in the ICU with HABSI have higher mortality rates compared to those without cirrhosis. Additionally, cirrhotic patients exhibit a higher prevalence of *E. faecium* HABSI compared to non-cirrhotic patients, suggesting the necessity for tailored empirical treatments. We concur with the reviewer's suggestion that further interventional research is warranted to investigate and validate these observations conclusively.

The conclusion section moderates our findings in line with the Reviewer comment: "In summary, critically ill cirrhotic patients with HABSI exhibit higher mortality rates than those without cirrhosis. They present a distinct microbiological profile, characterized by a predominance of Gram-positive bacteria, particularly *E. faecium* bacteremia. In light of these findings, empirical coverage of *E. faecium* should be considered in this specific patient population. However, the efficacy and safety of such antibiotic treatments necessitate confirmation through interventional studies designed specifically for this high-risk cohort."

2. Regarding the comment on the higher Rate of *E faecium* Compared to *E faecalis*:

The Reviewer rightfully points out the challenge in understanding the higher prevalence of *E faecium* compared to *E faecalis* observed in our study. Our hypothesis regarding antibiotic selective pressure remains speculative due to limited data on patients' prior antibiotic exposure, which was only collected for the last 7 days. However, cirrhotic patients are frequently exposed to antibiotics, especially for conditions like encephalopathy or peritonitis prophylaxis, and due to their higher incidence of infections, which supports this hypothesis.

We also point out in the manuscript that our population included only HABSI and this setting is characterized by higher prevalence of *E. faecium*, whereas E. *faecalis* tends to occur more frequently in the community-acquired setting (Page 10-11, lines 337-339)

Following the Reviewer comment, we now emphasize in the manuscript that this is hypothesis-generating only (Page 10, Line 328).

3. Regarding the acquisition setting of *E faecium* HABSI:

We appreciate the Reviewer's suggestion to analyze the acquisition setting of *E faecium* HABSI, and we have now conducted this analysis. In all HABSI cases, the majority were acquired in the ICU, with 75% among non-cirrhotic patients and 68% among cirrhotic patients (Table 2). Specifically focusing on *E faecium*, it was also predominantly acquired in the ICU, with 81.5% among non-cirrhotic patients and 90.5% among cirrhotic patients, with no statistically significant difference between the two groups. The observation that *E faecium* is primarily acquired in the ICU setting has now been highlighted in our manuscript (Supplementary Table 6, Page 8 of the Supplementary Materials, and main manuscript page 9 (Lines 299-300)), thanks to the reviewer's insightful comments.

It is worth noting that our multivariable model for the association between cirrhosis and *E faecium* HABSI was adjusted *a priori* for the place of acquisition of the HABSI. Despite this adjustment, cirrhosis remained independently associated with a higher risk of *E faecium* HABSI. This underscores the unique vulnerability of cirrhotic patients to *E faecium* infections.

| *E faecium* HABSI,  n=75 | Non cirrhotic  N=54 | Cirrhotic  N=21 | p |
| --- | --- | --- | --- |
| Site of acquisition, n (%)  -Ward  -ICU | 10 (18.5%)  44 (81.5%) | 2 (9.5%)  19 (90.5%) | 0.5 |

4. Regarding the Inclusion of Pulmonary Source of Infection in the Multivariable Logistic Mixed Model:

In the multivariable analysis, the variable "source" of infection was indeed included, encompassing all possible sources. This variable also includes lung and abdomen as a source of infection. Since the prevalence of pulmonary infection among non-cirrhotic patients is high, it is important to adjust for the source of infection (e.g., thus including pulmonary or abdominal sources of infection).

For Reviewer #1, we performed two supplementary analyses. 1) only including the binary variable abdominal source of infection. This supplementary analysis showed that cirrhosis remained significantly associated with *E. faecium* HABSI (OR 2.6, 95% CI 1.4-4.7, p<0.01). 2) A sensitivity analysis removing the source “lung”. This supplementary analysis showed that cirrhosis remained significantly associated with *E. faecium* HABSI (OR 2.5, 95% CI 1.3-4.8, p<0.01).

We would prefer to keep our original analysis in the manuscript.

Analysis 1) for Reviewer#1:

| *n=1059* | *E. faecium* BSI, Odds ratio (95%CI) | *p* value |
| --- | --- | --- |
| **Cirrhosis** | **2.6 (1.4-4.7)** | **<0.01** |

Results are adjusted for: reason for ICU admission, abdominal source of infection, location where the BSI was acquired, exposure to antibiotics in the last 7 days, delay (days) between hospital admission and BSI.

Results are expressed as odds ratios and 95% confidence interval (95% CI). A random effect for center was included.

Analysis 2) for Reviewer#1:

| *n=827* | *E. faecium* BSI, Odds ratio (95%CI) | *p* value |
| --- | --- | --- |
| **Cirrhosis** | **2.5 (1.3-4.8)** | **<0.01** |

Results are adjusted for: reason for ICU admission, source of infection, location where the BSI was acquired, exposure to antibiotics in the last 7 days, delay (days) between hospital admission and BSI.

Results are expressed as odds ratios and 95% confidence interval (95% CI). A random effect for center was included.

**Reviewer #2: My concerns have been addressed.**

**Reviewer #3: No more comment**

We thank Reviewers #2 and #3 for this review process and thoughtful feedback.
